# Side Effects of Dental Metal Implants: Impact on Human Health (Metal as a Risk Factor of Implantologic Treatment)

**DOI:** 10.1155/2019/2519205

**Published:** 2019-07-10

**Authors:** Jana Přikrylová, Jarmila Procházková, Štěpán Podzimek

**Affiliations:** Institute of Dental Medicine, First Faculty of Medicine, Charles University and General University Hospital in Prague, Kateřinská 32, Praha 2 121 11, Czech Republic

## Abstract

Dental implants are often made of titanium alloys. Implant therapy currently promises a good long-term result without impacting health; however, its success depends on many factors. In this article, the authors focus on the most common risk factors associated with metallic surgical implants. Titanium-induced hypersensitivity can lead to symptoms of implant rejection. Corrosion and biofilm formation are additional situations in which these symptoms may occur. For medical purposes, it is important to define and discuss the characteristics of metals used in implantable devices and to ensure their biocompatibility. To avoid hypersensitivity reactions to metallic dental implants, precautionary principles for primary prevention should be established.

## 1. Introduction

Dentistry is a continually evolving branch of medicine that is significantly affected by technological developments. The goal of modern implant dentistry is to restore physiological function, comfort, aesthetics, speech, and health to individuals who have missing teeth. Tooth loss is mostly caused by decay, by failed root canal treatment, by inflammatory loss of periodontal tissue, or by fracture [1, 2]. In the past, single tooth loss was usually treated with a three-unit fixed partial denture, filling the gap with a pontic which was supported on both sides by the abutment teeth. This treatment, also known as a fixed bridge, is not necessarily the optimal solution, as it requires crown preparation of the abutment teeth. As a result, these teeth are more susceptible to decay and gum disease, which can lead to further tooth loss or denture failure [2].

Unsightly gaps between teeth can be filled by dental implants without causing additional damage to other teeth [2]. Furthermore, endosseous implants can prevent the loss of alveolar bone. The alveolar processes, within the mandible and maxilla, surround and support the teeth to ensure their function. In contrast, chewing, biting, and speaking lead to micromovements of the tooth radix within its socket (periodontium), indirectly causing the rebuilding and remodeling of alveolar bone. When a tooth is lost, the lack of bone stimulation leads to decreased alveolar volume. As more teeth are lost, more areas of bone cannot be maintained [3]. An endosseous implant can prevent further bone loss but should be integrated into alveolar bone as soon as possible after extraction in order to prompt bone stimulation [4–6].

The above-mentioned advantages of endosseous prosthetics have attracted many dentists, resulting in the increased use of implant treatment. As the number of implants increases, it is necessary to continually focus on the biocompatibility of implant materials. In this article, the authors will review the most common risk factors associated with metallic surgical implants (i.e., corrosion, biofilm development, and hypersensitivity reactions) and then focus on the side effects that may arise in patients chronically exposed to metallic materials.

## 2. Mechanical and Chemical Properties of Titanium

Titanium is considered to be excellent biomaterial and the best choice for manufacturing permanent nonbiodegradable implants. Titanium is characterized by convenient mechanical properties such as its high strength-to-weight ratio, malleability, and low density. Titanium does not usually corrode [7] because it quickly becomes passive. Passivation involves the creation of an outer layer of shielding material that protects the bulk of the metal from the environment. Titanium oxidizes immediately upon exposure to air, forming a thin titanium dioxide (TiO_2_) layer, which quickly reforms if damaged, provided there is sufficient oxygen in the surroundings (self-healing effect) [8].

Titanium is nontoxic and infrequently rejected by the body. It has the inherent ability to osseointegrate, enabling its utilization as a dental implant material that can stay in place for several years. Findings in the literature, however, suggest that titanium can induce clinically relevant hypersensitivity reactions as well as other immune dysfunctions [9].

## 3. Corrosion

Corrosion is defined as the spontaneous and progressive loss of material and is caused by the surrounding environment [10]. Pure titanium is corrosion-resistant within controlled environments and in the absence of load [11]. However, under oral conditions and in combination with cyclic loads, titanium can corrode, thereby affecting the mechanical stability of the implant [12]. In addition, metallic debris produced after implantation may induce an enhanced inflammatory response or contribute to a hypersensitivity reaction [11]. There are many types of corrosion associated with metallic implants, such as galvanic, fretting, pitting, and crevice corrosion [10].

Galvanic corrosion often occurs when two different metallic devices are connected by an aqueous path (e.g., saliva), and it can greatly affect the mechanical stability and ultimate outcome of dental implants [10–12]. The basic unit of electrochemistry is the electrochemical cell, which is composed of an anode (titanium screw), a cathode (metallic fill), and an electrolyte (saliva). The electrodes connected in a circuit equalize their potential difference. Consequently, electrons are both generated and consumed. The current produced by the ion flow is used to measure the corrosion rate of a metal and is directly related to the material lost. The products yielded by corrosion may have cytotoxic or even neoplastic effects on the tissue surrounding the implant [10].

Fretting corrosion arises due to disruption of the protective layer on titanium screws. Pitting corrosion results from the spontaneous breakdown of the passivating film on a flat or overexposed area. Crevice corrosion is associated with uneven surfaces [12].

Corrosion of metallic implants may jeopardize the mechanical stability of the device, as well as the integrity of the surrounding tissue [11, 12]. Moreover, metal traces originating from implants have been proven to disturb homeostasis (e.g., DNA synthesis, mineralization, and mRNA expression of alkaline phosphatase). They have been found within the liver, the lungs, the lymph nodes, and the bloodstream. The electrical implications of corrosion on the surrounding tissue remain unclear [10]. However, it has been shown that electrical currents generated during corrosive events are amplified by cyclic loads (i.e., chewing; biting) [10, 11]. It is suggested that the surrounding tissues are chronically exposed to abnormal electrical signals [11].

## 4. Biofilm

Biofilms are a complex community of microorganisms attached to surfaces [13]. Biofilms may form on both living and nonliving surfaces and can be prevalent in natural (plant root system), industrial (water-heating system) [14], and hospital settings (catheters; implants) [15]. It is widely recognized that most bacteria coexist in association with surfaces. In other words, they do not usually live free-floating but tend to stick to each other and adhere to a specific surface [16]. They produce a slime (extracellular polymeric substances, EPS) consisting of nucleic acids, proteins, and polysaccharides [17]. EPS facilitates adhesion and serves as a nutrient for dividing microbes. It also provides communication between microorganisms through biochemical signals as well as the means for the exchange of genetic material [8]. Biofilms can be formed from a single species or mixed species [16]. Bacteria always interact and cooperate in many ways. Microbial cells growing in a biofilm differ from basal planktonic bacteria. Their structural and genetic development results in increasing resistance to disinfectants [13, 18]. In some cases, they are also resistant to antibiotics [19]. Furthermore, the final stage of biofilm formation is dispersal. The structure remains unchanged, but microbial colonies separate from the original biofilm, flowing away, and colonizing other surfaces [13]. Thus, biofilms can cause serious health problems and severe complications [15].

One example of an oral biofilm is dental plaque, consisting of both bacterial and mycotic species. It is anchored to the teeth within saliva polymers and should be removed by regular teeth cleaning. When oral hygiene fails, both the teeth (or dental implant) and the surrounding tissue (gum, periodontium, and alveolar bone) are subjected to high concentrations of microbial products, which can cause decay, gingivitis, periodontitis, or peri-implantitis. The above-mentioned conditions are not necessarily associated with the same microbial species; however, all potential oral pathogens may form an adherent population and grow within the dental plaque to subsequently trigger diseases of the oral tissues [20].

One of the greatest accomplishments in modern medicine has been the progress in curing infectious diseases. At present, most acute infections can be treated effectively with antibiotics. However, biofilms are an exception to this rule. Even after successful antibiotic therapy, the symptoms can recur very rapidly [21].

Implants serve as potential surfaces for the formation of biofilms. Surgical removal of implanted devices is the best cure for biofilm-related infections, but this is generally not considered to be an optimal solution [21]. To prevent biofilm formation, it is important to carefully follow all the rules of aseptic surgery [13]. Another method of decreasing the risk of bacterial adhesion is to develop new materials or to improve the surface of implanted medical devices so that they do not attract potential biofilm pathogens [15, 16]. Multifunctional coatings on a zirconia surface [22], a nanostructured titanium surface [23], and controlled antibiotic release may play a significant role in achieving this goal [15].

## 5. Metal Hypersensitivity

An allergy is defined as a hypersensitivity reaction. It is a disorder of the immune system, an overreaction to something that is usually harmless, but which triggers a reaction in anyone sensitive to the substance concerned [9].

Potential metal allergens are very common in daily life. The literature suggests that titanium can induce clinically relevant hypersensitivity in certain patients chronically exposed to this reactive metal. There are some obvious sources such as watches, jewellery, and coins; however, cosmetic products may contain sensitizing ingredients. Most patients with a titanium allergy are unaware of their exposure. For example, sunscreens containing TiO_2_ are applied on wide areas of the skin to avoid premature aging and skin cancer [24, 25].

If the allergen is ingested, one is likely to suffer from digestive symptoms. Inhalable particles affect the eyes, nose, and lungs [26]. Metal hypersensitivity can manifest as a range of adverse reactions, including chronic inflammation and pain. Common symptoms of metal hypersensitivity include chronic fatigue, depression, or fibromyalgia (pain without a known cause). The most common presentation of a patient with a metal allergy is a lichenoid reaction characterized by oral lichenoid lesions. These symptoms usually occur in patients with metal implants who are chronically exposed to metal allergens. However, metal implants can be rejected without any evidence of a previous hypersensitivity reaction [27].

Hypersensitivity is a sequence of undesirable reactions by the immune system. Their type and their development may differ. They are generally classified into four groups (I-IV) [28]. Metal hypersensitivity is usually a type IV hypersensitivity, known as a reaction of delayed hypersensitivity, because the reaction takes 48 to 72 hours to develop [24, 25, 28]. The first contact with a metallic antigen causes antigen-consuming (by Langerhans dendritic cells) and antigen-presenting to T_H_-lymphocytes. T_H_-lymphocytes have their origin in the thymus and are directed to produce cytokines that regulate the immune response pathways. If the patient is exposed to the metallic antigen again, the T_H_-lymphocytes activate macrophages [28]. Hence, inflammation is induced to counteract the perceived threat. The immune response may lead to tissue damage and eventually to aseptic implant loss [27].

Exposure to metals from dental endosseous implants, amalgam fillings, or joint prostheses can lead to serious health problems [24, 25, 27]. Reasonable suspicion of a metal hypersensitivity can allow for testing in sensitive patients, using the Memory Lymphocyte Immunostimulation Assay, known as the MELISA® test [24, 25, 29]. This is a blood test to detect metal hypersensitivity. T_H_-lymphocytes from blood samples are isolated and tested against selected metals, based on the patient's exposure. The stimulation index is used to measure a patient's reactivity to different metals. MELISA® is especially helpful in patients with symptoms of a metal allergy (chronic fatigue, chronic joint pain, contact dermatitis, and oral lichenoid reactions) but who have had negative patch tests [29].

Chronic problems may not occur until the implant has been put in place. It is very common to find traces of other metals (aluminium or nickel) in commercially pure titanium, as these substances contribute to better processability and prevent corrosion [25, 30]. Dental fillings, bridges, and implants can be potential sensitizers [24, 31], just like sunscreens, because they contain metals (nickel, aluminium, and titanium) [24, 25]. It is important to determine precisely which metals the patient is currently exposed to. Based on dental and medical treatments, as well as on environmental history, a list of all potential allergens should be established. To determine the problematic metal, it can be very helpful to remove other harmful agents (such as amalgam fillings and metal bridges) to decrease the potential number of metal-specific reactions [31]. An allergy evaluation for titanium is suggested in those titanium-implant-indicated patients who have a history of allergy to other metals [30].

It has been shown that people with a history of allergy to metals have a greater risk of developing a hypersensitivity reaction to a metal implant [32]. For sensitive individuals, there is no safe limit or acceptable level of a given metal substance [33]. Although titanium allergy has a low prevalence rate [9, 25], for patients with a previous history of allergies, it may be advisable to carry out a metal allergy assessment and allergy testing before placing permanent implants, in order to avoid a failure of the implant due to an allergic reaction to titanium [9, 30]. The MELISA® test is capable of defining which metals are tolerated in the sensitive patient and which induce an undesirable immune response [29, 33].

### 5.1. Management of Metal Hypersensitivity Reactions

Incidence of titanium sensitivity is increasing and its use in dentistry is increasing day by day as well [9]. The criterion standard for managing type IV hypersensitivity is avoidance of the responsible allergen [34].

Therefore, research has now focused on designing alternative substitutes to titanium [9]. Zirconium may be considered [34]. Oliva and colleagues reported a patient with amelogenesis imperfecta, who required full-mouth dental implants; titanium sensitivity was diagnosed based on elevated MELISA levels. Zirconium oxide implants and restorations were utilized with no complications at a 3-year follow-up [35]. One of other novel materials is Polyetheretherketone, which is a partially crystalline polyaromatic linear thermoplastic with excellent mechanical properties. Studies suggest that the implantable grade Polyetheretherketone has bone forming capacity comparable to rough titanium [9].

Successful medical management with oral atropine sulfate has been reported in a patient with titanium pacemaker [34] as well as with oral corticosteroids in a patient with titanium bioprosthesis for a spinal fracture [36].

If a patient with a titanium implant develops severe clinical symptoms strongly suggestive of titanium hypersensitivity, removal of the implant may be considered [37]. However, many dental implants (and other titanium implants) are intended to function for the remaining lifetime of the patient, and removal of the device may result in significant morbidity, loss of essential function, or even mortality. In these clinical scenarios, risks and benefits will need to be carefully weighed [34].

## 6. Conclusions

The advantages of endosseous prosthetics have attracted many dentists, resulting in the increased use of implant treatment. Increased life expectancy of the population demands the design of implant biomaterials demonstrating minimal deleterious effects on host tissues. Although traditional materials, such as titanium or its alloys have been widely used and promote osseointegration, there are some concerns such as metal ion release, allergic responses and biofilm formation. The definitive treatment for confirmed titanium hypersensitivity reaction is the removal of the device; however, medical management is possible in some cases. Better understanding of the risk factors associated with metallic surgical implants is necessary in patients undergoing dental implant treatment as well as joint replacement surgery.
